# *TNF*, *IL6*, and *IL1B* Polymorphisms Are Associated with Severe Influenza A (H1N1) Virus Infection in the Mexican Population

**DOI:** 10.1371/journal.pone.0144832

**Published:** 2015-12-14

**Authors:** Román Alejandro García-Ramírez, Alejandra Ramírez-Venegas, Roger Quintana-Carrillo, Ángel Eduardo Camarena, Ramcés Falfán-Valencia, Juan Manuel Mejía-Aranguré

**Affiliations:** 1 Coordination of Health Research, Mexican Institute of Social Security (IMSS), Av. Cuauhtémoc 330, 06720, Mexico, D. F., México; 2 Graduate Program in Biological Sciences, National Autonomous University of Mexico (UNAM), México, D. F., 04360, México; 3 HLA Laboratory, National Institute of Respiratory Diseases, Ismael Cosio Villegas, México, D. F., 14080, México; 4 Research Department on Smoking and COPD, National Institute of Respiratory Diseases, Ismael Cosío Villegas. Mexico, D. F., 14080, México; The Scripps Research Institute, UNITED STATES

## Abstract

**Background:**

Hypercytokinemia is the main immunopathological mechanism contributing to a more severe clinical course in influenza A (H1N1) virus infections. Most patients infected with the influenza A (H1N1) pdm09 virus had increased systemic levels of pro-inflammatory cytokines; including interleukin IL-6, IL-8, and tumor necrosis factor-alpha (TNF-α). We propose that single-nucleotide polymorphisms (SNPs) in the promoter regions of pro-inflammatory genes are associated with the severity of influenza A (H1N1) pdm09 virus infection.

**Methods:**

145 patients with influenza A (H1N1) (pA/H1N1), 133 patients with influenza-like illness (ILI), and 360 asymptomatic healthy contacts (AHCs) were included. Eleven SNPs were genotyped in six genes (*TNF*, *LT*, *IL1B*, *IL6*, *CCL1*, *and IL8*) using real-time PCR; the ancestral genotype was used for comparison. Genotypes were correlated with 27 clinical severity variables. Ten cytokines (GM-CSF, TNF-α, IL-2, IL-1β, IL-6, IL-8, IFN-γ, IL-10, IL-5, and IL-4) were measured on a Luminex 100.

**Results:**

The *IL6* rs1818879 (GA) heterozygous genotype was associated with severe influenza A (H1N1) virus infection (odds ratio [OR] = 5.94, 95% confidence interval [CI] 3.05–11.56), and two *IL1B* SNPs, rs16944 AG and rs3136558 TC, were associated with a decreased risk of infection (OR = 0.52 and OR = 0.51, respectively). Genetic susceptibility was determined (pA/H1N1 *vs*. AHC): the *LTA* rs909253 TC heterozygous genotype conferred greater risk (OR = 1.9), and a similar association was observed with the *IL1B* rs3136558 CC genotype (OR = 1.89). Additionally, severely ill patients were compared with moderately ill patients. The *TNF-238* GA genotype was associated with an increased risk of disease severity (OR = 16.06, p = 0.007). Compared with ILIs, patients with severe pA/H1N1 infections exhibited increased serum IL-5 (p <0.001) and IL-6 (p  =  0.007) levels.

**Conclusions:**

The *TNF* gene was associated with disease severity, whereas *IL1B* and *IL6* SNPs were associated with influenza A (H1N1) virus infection.

## Introduction

In mid-April 2009, the influenza A (H1N1) pdm09 virus emerged in Mexico and rapidly expanded across the globe. Worldwide, the mortality rate was approximately 0.2–1.23%; in Mexico, mortality was close to 0.4% [1]. The greatest number of deaths from influenza A (H1N1) pdm09 occurred in the central region of the country where healthcare systems are better than in the rest of the country and where access to healthcare services is greater because of better transportation routes and the presence of hospitals that provide care to patients with severe illnesses. However, the numbers of cases were similar in the northern and southern regions of the country [1]. The most affected groups were individuals aged 30–46 years [2], and a high percentage had no comorbidities that could explain the most severe forms of the disease.

Hypercytokinemia ("cytokine storm") is the main immunopathological mechanism underlying a more severe clinical course in cases of seasonal influenza and is the ultimate cause of death [3]. Most patients infected with the influenza A (H1N1) pdm09 virus have increased serum levels of pro-inflammatory cytokines, such as interleukin IL-6, IL-8, IL-1β and tumor necrosis factor-alpha (TNF-α) [4–7]. In particular, elevated IL-1β and IL-6 serum levels have been identified as markers of severity in acute lung injury during infection by the influenza A (H1N1) pdm09 virus, specifically among patients who do not respond to conventional antiviral treatments [8, 9]. Multiple cytokines are associated with the pathophysiology of inflammation and lung remodeling. Several reports from around the world have shown the presence of an imbalance of pro-inflammatory cytokine responses in severe H1N1 pneumonia [10, 11]; however, little is known about why these differences occur.

Cytokine production varies among individuals due in part to genetic factors and, particularly, the presence of polymorphisms in important regulatory regions such as promoters [12, 13]. In this study, the differential expression of cytokines among individuals with influenza pA/H1N1, those who developed a clinical picture similar to influenza but were not positive for the pandemic virus, and AHCs was examined.

In a previous study performed by our team, several single-nucleotide polymorphisms (SNPs) associated with the risk of influenza infection, including *CCL1* rs2282692, *LTA* rs909253, and *TNF* 1800750, were identified in a population in Mexico City. [2] Other groups have reported associations between SNPs in pro-inflammatory cytokines and disease severity [14–16].

According to *Borja et al*. *2012*, the fourth-wave pattern of influenza A (H1N1) observed in Mexico during the 2011–2012 season was not reported in other countries. These differences may be explained by vaccination coverage or genetic susceptibility in the Mexican population [17]. In this study, we included a greater number of patients from the fourth wave of virus infection and aimed to establish the association between polymorphisms in inflammation-related genes (*TNF*, *IL6*, *IL8*, *IL1B*, *LTA*, *CCL1*) and disease development, severity and death from influenza A (H1N1) virus infection in patients from the central region of Mexico.

## Materials and Methods

This case-control, ambispective study was performed from April 2009 to March 2012 using nasopharyngeal swab samples from 278 patients hospitalized with clinical symptoms of influenza-like illness.

### Ethics Statements

This study was reviewed and approved by both the Bioethics Committee and Science Committee in Research, with protocol number B05-10 at INER (National Institute of Respiratory Diseases Ismael Cosio Villegas) and Institutional Review Board No. 13CI 09015 213 at IMSS (Mexican Institute of Social Security) authorized by COFEPRIS/SSA.

A trained and responsible physician informed the patient about a possible diagnosis of influenza-like illness (ILI). All participants were invited to participate voluntarily by a trained researcher and gave signed informed consent prior their enrollment. All patients received medical care even if they chose not to participate in the research protocol. The signed consent forms and ethics committee approval letters are documented at HLA Laboratory INER. Data were obtained using clinical records according to the Mexican Official Standard NOM-168-SSA1-1998. A clinical investigation was conducted according to the principles expressed in the Declaration of Helsinki. The ethics committees described above approved our data recording procedures.

### Diagnosis

All patients underwent the QuickVue Influenza A + B test (*Quidel*, *San Diego*, *CA*, *USA*). Additionally, peripheral blood from patients and asymptomatic healthy contacts (AHC) was collected; these interventions adhered to the guidelines and recommendations of the Centers for Disease Control and Prevention of the United States (CDC) and the World Health Organization (WHO). Subsequently, the RespiFinder assay was administered, and the presence of influenza A (H1N1) virus was confirmed.

### Population

All subjects included in our study were evaluated according to a clinical questionnaire, which contained questions regarding influenza vaccination. The inclusion period was from May 2009 to March 2012, so the subjects examined during the study belonged to three different waves of influenza in Mexico.

A total of 278 subjects with clinical symptoms and signs of influenza were enrolled, of which 145 were positive and 133 were negative for influenza A (H1N1) infection; the influenza-negative patients were deemed to have ILI. On admission to the hospital, all patients with suspected influenza were treated with oseltamivir.

**Fig 1:**
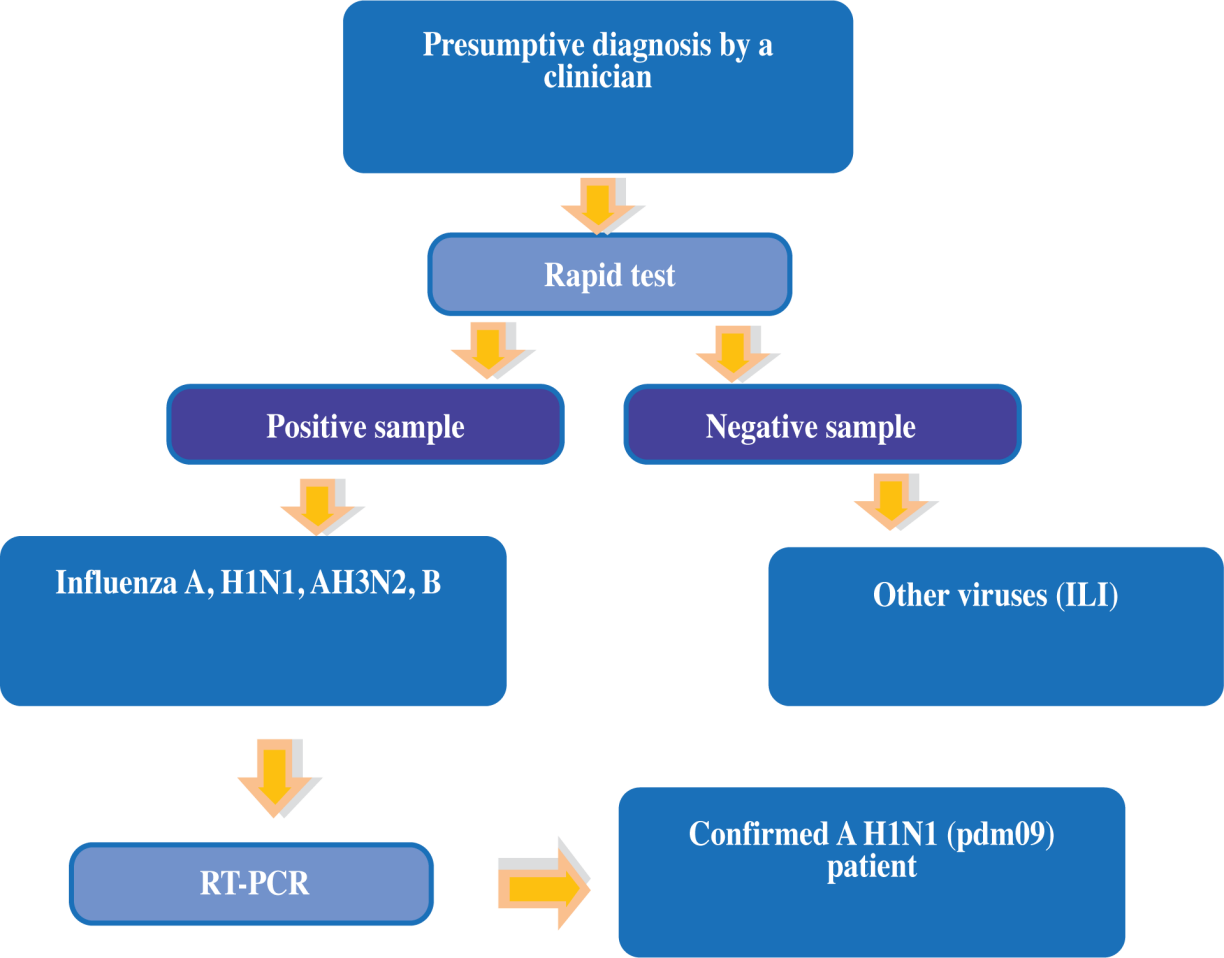
Flowchart for the collection and classification of patient samples in the study. The blood samples were collected from suspected influenza inpatients at INER from 2009–2012. Abbreviations: ILI: influenza-like illness; RT-PCR: real-time PCR.

In this study, most patients sought medical attention 3–4 days after the onset of symptoms. All clinical data were collected retrospectively; nonetheless, blood samples and nasopharyngeal swabs were collected at the hospital during the first 24 hours after admission. In the group of AHCs, 360 biologically unrelated, intra-household contacts were enrolled who were asymptomatic and in close, continuous contact with ILI or H1N1 patients before and during admission to the hospital (Fig 1). None of the AHCs developed symptoms or was hospitalized for influenza virus infection. Peripheral blood from these individuals was tested for anti-A (H1N1) antibody titers by hemagglutination inhibition assay according to the method described by Julkunen I, 1985 [18] to determine exposure to the virus. All subjects included in this group had HI titers greater than 1:16, indicating direct contact with the virus. All pA/H1N1 subjects were less than 60 years of age, precluding any cases of cross-reactive antibodies, which are prevalent in older age groups due to H1N1/1918-1919 infection [19]. Because this cohort was not progressively monitored for the dynamics of anti-A H1N1/09 antibody titers over time, these results are not shown.

### Data Collection and Variables

The study protocol was explained to all participants, and authorization was obtained with an informed consent form. The information collected included demographic data as well as laboratory data, medical histories, and drug treatment records.

### Genotyping of Allelic Variants (SNPs)

DNA samples were genotyped for *TNF* rs361525, rs1800629, and rs1800750; *LTA* rs909253; *IL1B* rs16944, rs3136558, and rs2227307; *IL6* rs1818879, rs2069840, and rs2066992; and *CCL1* rs2282691 polymorphisms using commercial TaqMan assays (Applied Biosystems, CA, USA). The genetic data for the SNPs are described in S1 Table. The polymorphisms were characterized by real-time PCR allelic discrimination on a 7300 Real-Time PCR System using 15 ng of DNA. The amplification conditions were as follows: 50°C for 2 min and 95°C for 10 min followed by 40 cycles of 95°C for 15 s and 60°C for 1 min.

Haploview version 4.2 was used to determine the presence of haplotypes in *IL6*, *IL1B* and *TNF* genes associated with the risk of both H1N1 viral infection and greater clinical severity.

### Cytokine Quantification

Serum samples were taken from patients with ILI or a suspected ILI diagnosis upon hospital admission. The enrolled patients and the healthy volunteers gave whole blood, which was allowed to clot for 30 minutes at 37°C and was stored thereafter at -70°C until use. The resulting serum was used for cytokine measurement. Ten cytokines (GM-CSF, TNF-α, IL-2, IL-1β, IL-6, IL-8, IFN-γ, IL-10, IL-5, and IL-4) were measured on a Luminex 100 (Luminex Corporation, Austin, TX, USA) using a multiplex cytokine kit as well as the Cytokine Human 10-Plex Panel (Novex, Life Technologies, CA, USA) in accordance with the manufacturer's instructions.

### Statistical Analysis

Differences between the group proportions were analyzed using the chi-square test. Differences between clinical parameters, continuous variables, and each polymorphism were analyzed using the Mann-Whitney U test. The ancestral genotype was used as a reference for each polymorphism. Odds ratios (ORs) were calculated with 95% confidence intervals (CIs). The ORs were adjusted for age and disease severity in the logistic regression model. SPSS 21 (Chicago, IL, USA) and Epi-Info 7.0 (CDC, GA, USA) were used for these analyses.

For cytokine analysis, the subjects were stratified into three groups: 18 patients with A (H1N1) influenza, 15 patients with ILI, and 21 AHCs. Descriptive statistics included means and standard deviations or medians and interquartile ranges for continuous variables with normal and non-normal distributions, respectively. The Fisher exact test and the chi-square test were used for categorical variables. The Mann-Whitney U test was used for nonparametric variables. The nonparametric Wilcoxon test was used to compare two paired groups. The association between nonparametric variables was determined using the Spearman correlation coefficient (r). Any value of p<0.05 was considered statistically significant. GraphPad Prism version 5.03 for Windows (GraphPad Software, La Jolla, CA, USA) was used for these analyses.

## Results

### Demographic Characteristics

This study compared three groups: patients with confirmed influenza A (H1N1) (pA/H1N1), subjects with influenza-like illness (ILI), and AHCs. Gender distributions were similar in the three groups of patients, and differences between the groups were not significant. Patients in the pA/H1N1 group were slightly younger (42±14 years) than those in the ILI group (45±15 years, p = 0.068). In addition, the presence of comorbidities was assessed, including diabetes, hypertension, asthma, and chronic obstructive pulmonary disease (COPD); interestingly, in the pA/H1N1 group, there was a higher rate of diabetes than in the ILI group (12.5% *vs*. 5.21%, respectively; p = 0.03). There were no statistically significant differences for the other comorbidities studied (Table 1).

**Table 1:** Characteristics of the patients at hospital admission.

Variable	pA/H1N1	ILI	p
	(n = 145)	(n = 133)	
Sex (M/F, %)	64.75/35.25	64.95/35.05	
Age (years)	42±14	45±15	
Height (cm)	164 (155–170)	165 (153–171)	
Weight (kg)	81±24	78±20	
BMI	30±7	28±5	0.0062
HR	90 (76–110)	92 (79–106)	
RR	24 (20–28)	22 (20–25)	0.0363
FA	101 (76–127)	88 (73–135)	
PaO_2_	51 (42–62)	54 (48–68)	0.024
PaCO_2_	30 (27–33)	30 (28–35)	
Clinical variables			
Leukocytes	7 (5–10)	9 (6–12)	0.0005
Neutrophils	8 (5–63)	10 (7–67)	
Platelets	175 (135–241)	221 (156–267)	0.0067
Glucose	123 (103–157)	107 (95–128)	0.0005
BUN	14 (10–19)	13 (9–18)	
Urea	29 (20–41)	28 (19–40)	
Creatinine	0.87 (0.67–1.05)	0.79 (0.62–1.03)	
CPK	245 (112–522)	149 (77–320)	0.0047
LDH	657 (417–1.069)	379 (213–655)	<0.001
AST	56 (33–87)	38 (25–63)	0.001
ALT	41 (27–75)	36 (22–58)	0.0185
Comorbidities			
Diabetes (Y/N, %)	12.5/87.5	5.21/94.79	
Hypertension (Y/N, %)	16.79/83.21	17.71/82.29	
Asthma (Y/N, %)	8.76/91.24	14.58/85.42	
COPD (Y/N, %)	1.47/98.53	3.13/96.88	
Complications			
ICU % (Y/N)	52.99/47.01	16.84/83.16	<0.001
AMV % (Y/N)	46.72/53.28	17.71/82.29	<0.001
ARDS % (Y/N)	46.72/53.28	17.71/82.29	<0.001
Pneumonia % (Y/N)	90.37/9.63	73.96/26.04	0.001
Death % (Y/N)	31 (22.46)	4 (4.21)	<0.001

Clinical differences among pA/H1N1 *vs*. ILI. Demographics, clinical records, complications, and major comorbidities in both comparison groups: pA/H1N1 *vs*. ILI inpatients from 2009–2012 influenza waves. HR, RR, FA, PaO_2_, PaCO_2_ and clinical variables are presented as medians and interquartile ranges; other demographic variables are presented as the mean ± standard deviation, and p-values were derived using Student’s t test. Comorbidities and complications are expressed as percentages. Abbreviations: pA/H1N1: patients with influenza A (H1N1); ILI: patients with influenza-like illness; BMI: body mass index; HR: heart rate; RR: respiratory rate; ICU: intensive care unit. AMV: mechanical ventilation; ARDS: acute respiratory distress syndrome; PaO_2_: oxygen partial pressure; PaCO_2_: carbon dioxide partial pressure; Y/N: yes/no.

Patients with confirmed influenza A (H1N1) consistently showed a greater disruption of clinical parameters influencing evolution toward greater disease severity (Table 2).

**Table 2:** Differences in clinical parameters of severity among patients with confirmed influenza pA/H1N1 *vs*. ILI.

Variable	pA/H1N1 (%)	ILI (%)	p
Platelets	121 (100)	88 (100)	0.009
<150,000/mm^3^	40 (33.06)	15 (17.05)	-
Glucose	116 (100)	80 (100)	<0.01
>119 mg/dL	67 (57.76)	25 (31.25)	-
CPK	116 (100)	79 (100)	0.021
>240 U/L	59 (50.86)	27 (34.18)	-
LDH	120 (100)	87 (100)	0.035
>1200 U/L	22 (18.33)	7 (8.05)	-
AST	121 (100)	79 (100)	0.009
>63 U/L	51 (42.15)	19 (24.05)	-

Clinical parameters comparing the percentage of subjects in each group with severity variables; cutoffs for each clinical parameter were determined according to international standards. N (%). chi-square test. Abbreviations: OR: odds ratio; CI: confidence interval.

### Risk of Severe Influenza A (H1N1) Infection

To determine the risk of severe influenza A (H1N1) virus infection, the genotypes of individual SNPs were compared in both groups of patients (pA/H1N1 and ILI).

Genotypes associated with influenza A (H1N1) virus infection were identified. The *IL6* rs1818879 (GA) heterozygous genotype showed a risk association with viral infection (p<0.001; OR = 5.94, 95% CI: 3.05–11.56). Two *IL1B* SNPs were associated with a decreased risk of infection: rs16944 AG (OR = 0.52, 95% CI: 0.28–0.97) and rs3136558 TC (OR = 0.50, 95% CI: 0.29–0.88) (Table 3).

**Table 3:** Genotype frequencies and associations with susceptibility and risk of severe influenza A (H1N1) infection.

Gene/SNP	Location	Genotype	pA/H1N1	ILI	AHC	pA/H1N1 *vs*. AHC			pA/H1N1 *vs*. ILI		
	Region		GF (%)	GF (%)	GF (%)	p	OR	95% CI	p	OR	95% CI
***TNF***											
rs361525	-238 (Promoter)	GG	88.9	88.6	88.5						
		GA	9.7	9.8	10.1						
		AA	3.4	1.5	1.4						
rs1800629	-308 (Promoter)	GG	93.1	92.4	92.6						
		GA	6.9	4.6	7.1						
		AA	0	3.1	0.3						
rs1800750	-376 (Promoter)	GG	95.8	95.5	96.9						
		GA	4.2	1.5	3.1						
		AA	0	3	0						
***IL1B***											
rs16944	-511 (Promoter)	AA	34.3	31.8	31.4						
		AG	46.2	33.3	4.4						
		GG	19.6	34.8	24.6				4.3E-02	0.52	0.28–0.97
rs3136558	Intronic	TT	36.2	23.8	32.6						
		TC	44.9	58.5	58.5				1.7E-02	0.50	0.29–0.88
		CC	18.8	17.7	8.9	4.10E-02	1.89	1.02–3.51			
***IL6***											
rs18181879	3´ UTR	GG	22.1	45.1	22.5						
		GA	47.9	16.4	46.8				5.0E-08	5.94	3.05–11.56
		AA	30	38.5	30.7						
rs2069840	Intronic	CC	50.7	41.7	46.5						
		CG	40.3	47	45.6						
		GG	9	11.4	7.9						
rs2066992	Intronic	GG	65.5	59.8	50.1						
		GT	34.5	39.4	49.6	2.0E-03	0.53	0.35–0.80			
		TT	0	08	03						
***CCL1***											
rs2282691	Intronic	TT	39	32.5	33.1						
		TA	39	39.7	40.1						
		AA	22	27.8	26.7						
***IL8***											
rs2227307	+396 (Intronic)	TT	38.9	4.7	42.3						
		TG	43.9	40.9	46.5						
		GG	11.8	12.1	11.2						
***LTA***											
rs909253	+252 (Intronic)	GG	40.7	40.2	54.3						
		GA	48.3	50	34	3.0E-03	1.9	1.25–2.87			
		AA	11	9.8	11.7						

Estimated associations among genotypes to determine the risk of severe influenza A(H1N1) infection (pA/H1N1 *vs*. ILI)and disease susceptibility (pA/H1N1 *vs*. AHC). Only statistically significant data are shown. Abbreviations: pA/H1N1: patients with influenza A (H1N1); ILI: patients with influenza-like illness; AHC: asymptomatic healthy contacts; GF: genotype frequency; UTR: untranslated region; OR: odds ratio; CI: confidence interval.

### Disease Susceptibility

When the pA/H1N1 group was compared with the AHC group, we observed that the *LTA* rs909253 TC heterozygous genotype showed a risk association with disease susceptibility (p = 0.003; OR = 1.9, 95% CI: 1.25–2.87); a similar association occurred with the *IL1B* rs3136558 CC genotype (p = 0.041; OR = 1.89, 95% CI: 1.02–3.51). In contrast, the *IL6* rs2066992 GT genotype was protective (p = 0.002; OR = 0.53, 95% CI: 0.35–0.80) for individuals carrying the homozygous genotype for the ancestral allele (Table 3).

### Susceptibility to Greater Severity of A (H1N1) Infection

To determine the susceptibility to greater disease severity in relation to the SNPs present in pro-inflammatory genes, two subgroups were compared: patients admitted to the ICU and those who did not enter the unit and therefore did not require AMV; including both pAH1N1 patients and ILI patients (53%vs. 17%). Of the 11 SNPs studied, the *TNF* rs361525 SNP was associated with greater disease severity (p = 0.007; OR = 16.06) (S3 Table).

### Allele Analysis

With respect to *LTA* rs909253, allele A is a risk factor for disease development (pA/H1N1 *vs*. AHC: p = 0.049; OR 1.34, 95% CI: 1.01–1.80), whereas the *IL6* rs2066992 T allele is a protective factor (pA/H1N1 *vs*. AHC: p = 0.007; OR = 0.62, 95% CI: 0.43–0.98). In addition, the *IL1B* rs16944 G allele is protective against the development of severe influenza A (H1N1) virus infection (pA/H1N1 *vs*. ILI: p = 0.048; OR = 0.70, 95% CI: 0.50–0.98) (S2 Table).

### Mortality

Thirty-one patients positive for influenza A (H1N1) virus died, whereas four patients with ILI died. None of the polymorphisms/genotypes were directly associated with mortality. We also aimed to demonstrate whether there was any additional risk besides mortality associated with the presence of comorbidities; only the *IL6* rs2069869 GG genotype was associated with mortality risk in patients with diabetes in the pA/H1N1 group (p = 0.011; OR = 40.8, 95% CI: 2.31–720.24). Only statistically significant data are shown.

### Haplotypes

**Fig 2:**
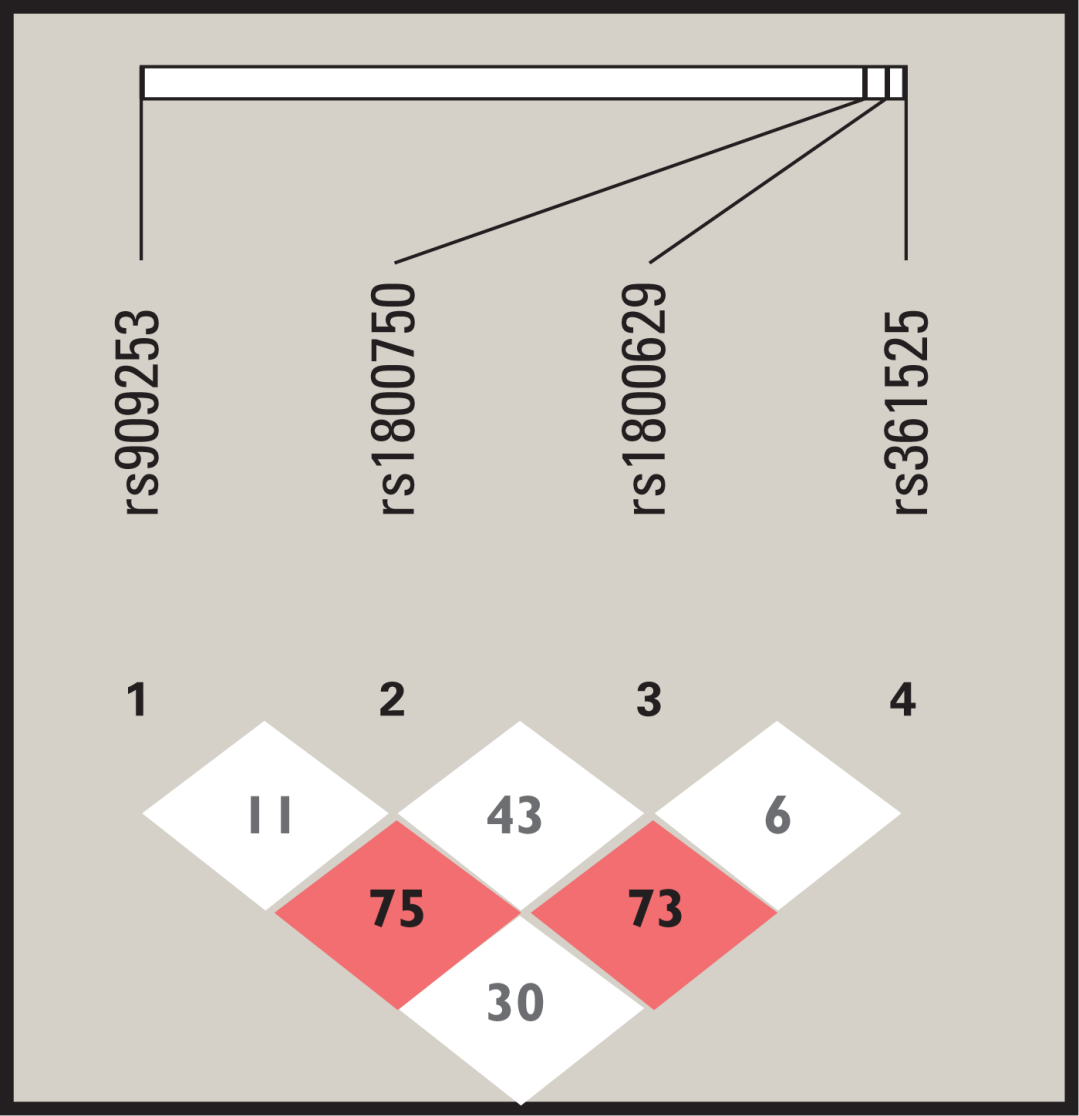
Haplotype for TNF gene at Chr 6 in ILI patients. Linkage disequilibrium between the four single nucleotide polymorphisms identified at chromosome 6 in the TNF gene (generated for the ILI patients in Haploview 4.2).

The haplotype study was conducted to determine their associations with disease severity. The analysis included the *IL6*, *TNF*, and *IL1B* genes with their respective SNPs and considered both the pA/H1N1 group *vs*. the *ILI* group and each group individually. When comparing the two groups of patients, haplotypes were not formed, only independent alleles, in groups of patients (ILI or pA/H1N1). Fig 2 shows the haplotypes consisting of the *TNF* SNPs located on chromosome 6 in the patients with ILI; these haplotypes are not in high linkage disequilibrium (r^2^ = 0.75), although the value was close to the minimum required (0.80). Polymorphisms evaluated in the *TNF* and *IL6* genes met Hardy-Weinberg equilibrium (Fig 2).

### Clinical Severity

Five parameters of infection severity were considered: mechanical ventilation, admission to ICU, presence of Acute Respiratory Distress Syndrome, development of pneumonia, and fatal outcome.

The analysis of severity variables confirmed that these variables *per se* are significantly associated with disease severity (Table 3). Interestingly, associations were identified between the values for lymphocytes <1,000 and glucose >119 mg/dL with the rs16944 and rs1818879 GG genotypes in *IL1B* and *IL6*, respectively. Additionally, the rs16944 GG genotype was associated with thrombocytopenia and the rs1818879 GG genotype with the use of AMV (Table 4).

**Table 4:** Genotypes associated with clinical severity variables in patients with influenza A (H1N1) pdm09.

Clinical severity	p	OR	95% CI
*IL6* rs1818879 GG			
Glucose >119 mg/dL	0.0035	4.8	1.48–15.57
Lymphocytes <1 x 10^3^ /L	0.0003	3.1	1.59–16.18
Mechanical ventilation	0.0004	4.8	1.29–18.24
*IL1B* rs16944 GG			
Glucose >119 mg/dL	0.0068	3.1	1.02–9.49
Lymphocytes <1 x 10^3^ /L	0.0118	2.3	1.13–4.51
Platelets <150,000/ mm^3^	0.0063	8.3	1.77–39.43

Logistic regression was performed to correlate these severity cutoffs with the 11 polymorphisms studied. Only statistically significant data are shown. Abbreviations: OR: odds ratio; CI: confidence interval.

### Serum Cytokine Expression

**Fig 3:**
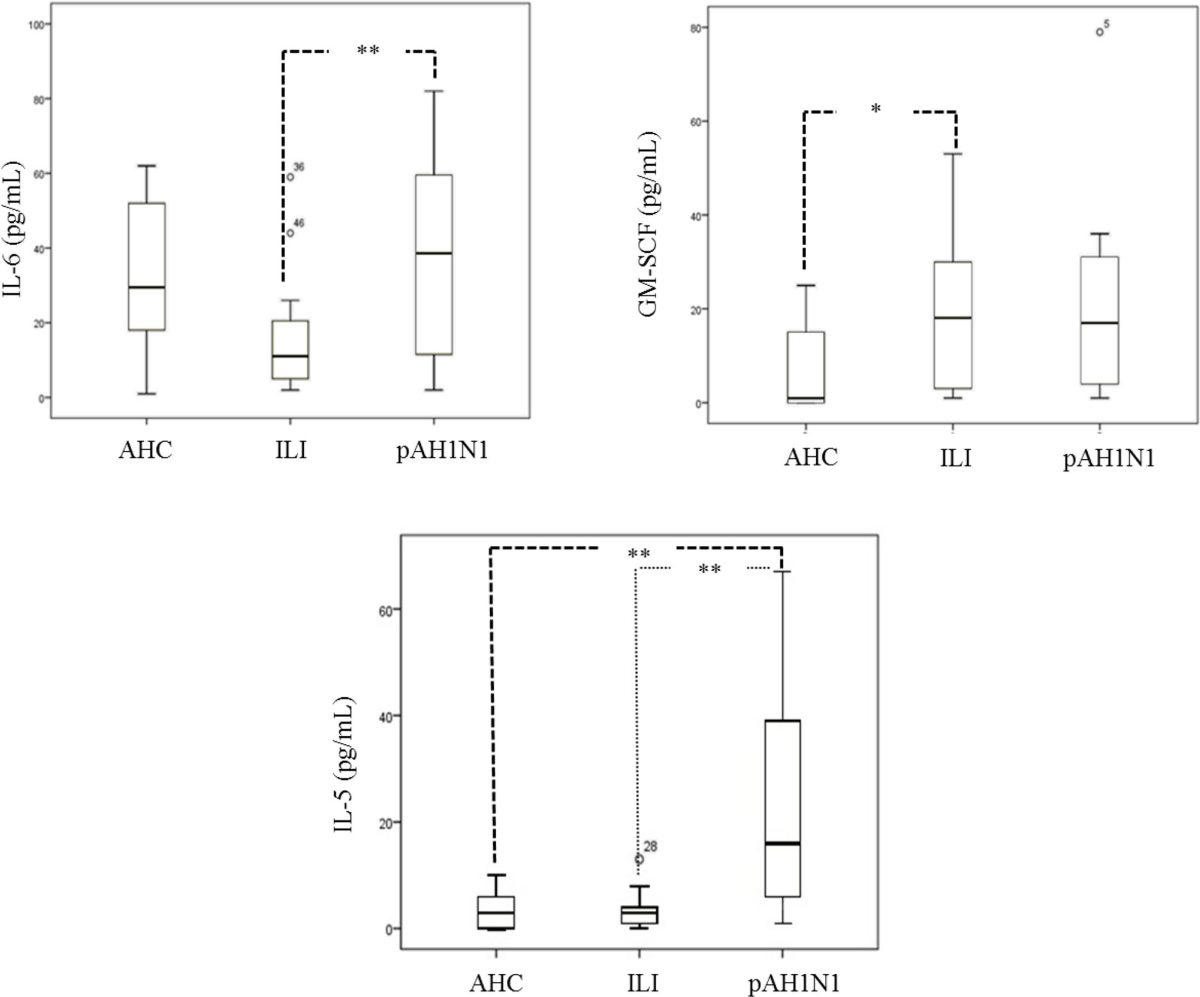
Concentrations of IL-6, IL-5, and GM-SCF in p AH1N1, ILI patients and AHC at admission. Box and whisker plots of cytokine levels in sera from the AHC, ILI, and pA/H1N1 groups. Boxes represents the 25–75 quartile, the whiskers represent the range of values, the middle line inside the box is the median, and the outlier values are represented as circles outside the boxes. The p-values (** and * representing <0.001 and <0.05, respectively) were calculated by one-way ANOVA and are presented above the graphs.

We determined the levels of ten diverse pro-inflammatory cytokines in serum samples. Because the cytokine concentrations showed significant inter-individual variation, we represented the quantification in a box plot in Fig 3. The concentrations of cytokines were in the range of 5–80 pg/mL. In the case of pA/H1N1 patients, the concentrations of all cytokine were higher than in the other study groups. In particular, IL5 showed increased concentrations in pA/H1N1 patients *vs*. ILI patients or AHCs. The three groups were compared using one-way ANOVA for multiple comparisons. All comparisons were considered statically significant at p<0.01 and p<0.05; for Tukey corrections, p<0.03 was considered significant. For IL6, the multiple comparisons ANOVA model produced a marginal p = 0.03.

Compared with ILI patients, severe pA/H1N1 patients exhibited markedly increased levels of serum IL-5 (p <0.001) and IL-6 (p = 0.007) and similar levels of GM-CSF (p =  0.967). We found no statistically significant differences for the remaining seven analytes evaluated. These results show that severe pA/H1N1 patients secrete higher levels of serum IL-6 and IL-5 than do ILI patients, suggesting a particular mechanism of pathophysiology in pA/H1N1 infection.

Although the levels of serum IL-6 in pA/H1N1 patients were higher than those of healthy controls, no statistically significant differences were observed (Fig 3).

Finally, we examined the correlation between cytokine levels and severe clinical parameters, including obesity and leukocyte counts. The median IL-5 concentrations for obese ILI patients and for obese pA/H1N1 patients were 3.08 and 3.07, respectively. In contrast, compared with non-obese ILI patients, non-obese pA/H1N1 patients showed a 9-fold increase: median of 3.03 *vs*. median of 29.93 (p<0.01), respectively. Concentrations of IL-6 in obese ILI patients and in obese pA/H1N1 patients were 10.58 and 32.30, respectively. Compared with non-obese ILI patients, non-obese pA/H1N1 patients showed an increase: 19.41 *vs*. 49.17; however there was no significant difference (p = 0.28).

Pearson’s analysis showed no significant difference between BMI and IL-5 and IL-6 concentrations or leukocyte counts and IL-5 and IL-6 in the pA/H1N1 group.

## Discussion

In addition to the findings previously described, in this second study of polymorphisms in pro-inflammatory cytokine genes, our group identified genetic factors associated with susceptibility to influenza A (H1N1). In late 2012, we reported that some genotypes in *TNF* and *LTA* are associated with susceptibility to infection with the influenza A (H1N1) pdm09 virus. Interestingly, two SNPs, one in *IL1B* (rs16944) and one in *IL6* (rs1818879), were associated with clinical severity when comparing influenza A (H1N1) patients with ILI patients.

This study confirms our previous finding that the *LTA* rs909253 GA genotype is a risk factor for susceptibility to developing secondary disease after contact with the influenza A (H1N1) pdm09 virus; interestingly, the minor allele (A) was associated with risk in this same comparison group (pA/H1N1 *vs*. AHC).

To complement these observations, an association analysis was conducted regarding the risk of severe influenza A (H1N1) infection; both *IL1B* and *IL6* SNPs showed significant associations, particularly rs16944, which is located in the promoter region of the gene. In agreement with what has been reported previously, the GA genotype in the *TNF*-238 (rs361525) promoter presented a risk for greater disease severity in patients who were admitted to the ICU compared with those who only required hospitalization [2, 15, 20]. Despite having no association with the minor allele homozygous genotype for *TNF*-238, we may observe that the presence of allele A in the heterozygous genotype in severe influenza A (H1N1) patients increased the risk of severity.

Associations have recently been reported for the *IL1A*, *IL1B* [21] and *IL10* [16] genes, whereas increases in the allelic frequencies of *TNF*-238A (rs361525) and *TNF*-308G (rs1800629), *IL10*-592C, and *IL10*-1082A have been associated with increased disease severity [14–16].

Both *LTA* and *TNF* are considered biologically relevant inflammatory cytokines with various functions in the immune response. Several authors have shown that SNPs present in these genes are associated with increased susceptibility for developing infectious diseases [22–24]. The polyadenylation region in *LTA* and the *TNF* transcription start sites are in close proximity in the *TNF* cluster of the HLA class III region; therefore, it is difficult to obtain associations of SNPs in this region [25]. Currently, priority is given to studies of haplotypes for candidate immune response genes, especially for multigenic control in response to infectious diseases such as influenza. Therefore, we conducted a study of haplotypes in influenza A (H1N1) and ILI patients; however, a haplotype between *LTA* and *TNF* was only identified in the latter group, and high linkage disequilibrium was not observed (r^2^ = 0.75). We believe that expanding the *n* or considering other candidate genes could improve this approach. Because cytokine production is influenced by SNPs in the promoter regions of these genes [26], we propose that individuals carrying associated alleles may exhibit increased cytokine production and may thus develop more severe disease.

Previous studies have reported a correlation between IL-1β and IL-6 overexpression and some clinical, radiological and immunological findings, such as pneumonia and ARDS, in both children with seasonal influenza and murine models [27–29]. TNF, IL-1β and IL-6 pro-inflammatory cytokines play crucial roles in inflammation, infection, and responses to stress caused by different types of infections. Many of the systemic effects of the acute phase are due to the combined actions of these cytokines [30]. In particular, IL-1β and IL-6 have been identified as markers of severity in acute lung injury during influenza A (H1N1) virus infection [31]; IL-1β in particular has been proposed as a good early marker of the severity and progression of lung inflammation in mechanically ventilated patients [9]. In a 2013 study by *Chiaretti et al*., influenza A (H1N1) virus infection was shown to induce the early and significant up-regulation of IL-1β and IL-6 in plasma, suggesting that these cytokines are responsible for different molecular reactions that lead to inflammation of the airway and increased disease severity [32]. In turn, IL-6 plays a critical role in the acute phase response during infectious processes and is usually induced with other pro-inflammatory cytokines, such as TNF-α and IL-1β, during immune system alarms [33]. Similarly, these cytokines are overexpressed at higher serum levels in severe patients compared with patients with moderate disease [4–7]. Strikingly, in our study, compared with obese pA/H1N1 patients, non-obese patients showed significantly higher IL-5 and IL-6 levels; furthermore, pA/H1N1 patients exhibited lower leukocyte counts. Several groups worldwide have reported that increased levels of pro-inflammatory cytokines are associated with clinical severity parameters, including obesity [34,35]. We found that IL-6 levels were higher in pAHN1 obese patients than in ILI obese patients (32.3 *vs*. 10.5 pg/mL), with no significant differences. In contrast, non-obese patients in the pA/H1N1 group showed a 9-fold increase in IL-5 levels. Despite the increase observed in both obese patient groups, it is important to note that compared with ILI patients and AHCs, non-obese patients with severe clinical pictures of H1N1 influenza have increased IL-5 levels. There were no significant differences for leukocyte counts and cytokine levels in pA/H1N1 and ILI patients.

According to published reports, cytokine levels are affected over time [10, 36]. Nevertheless, our study only examined associations between SNPs and susceptibility to infection and disease severity; we did not investigate how oseltamivir affects cytokine levels over time. Oseltamivir treatment may modify the expression of cytokines, but that does not affect the association between certain SNPs and disease severity among patients and AHCs. The median number of days from the onset of symptoms to oseltamivir administration was 5.0, although 92% of study patients were administered oseltamivir within 1 day after admission [37]. Therefore, associations were analyzed by the severe clinical picture regardless of immune regulation by the antiviral, which was reinforced by the fact that the patients were subjected to the same doses and began treatment similarly. One major limitation of this study was that samples were only collected in the central region of the country where mortality was higher; therefore, we believe that expanding the sampling area to other geographical regions would enable us to clearly identify areas with greater "genetic vulnerability" to be able to overcome future challenges of potentially lethal subtypes of influenza.

Concerning age at presentation, our results indicate that many patients who are not in the age groups at greatest risk of death or who have no comorbidities associated with an increased risk of death from influenza may have genetic characteristics that make them more susceptible to developing influenza and presenting with greater disease severity. With respect to the population of 2010 and 2012 and in accordance with what has been reported by other authors [38–40], approximately 95% of our study population had not previously received an influenza vaccine due to the vaccination guidelines suggested by the Ministry of Health prior to 2013. In addition to this intricate scenario, many patients are reluctant to receive vaccines due to mistrust of the vaccine or infection with H1N1 [41]. In this way and based on our exclusion criteria, >90% of our study population was excluded from the risk group. We reasoned that associations with the severity of influenza A (H1N1) virus infections would be determined more by genetic variables than an immune response to the influenza vaccine. This view is supported by the fact that the group of AHCs is in the same age group and therefore has the same vaccination characteristics. Therefore, it is unlikely that our results would be modified by vaccination status.

## Conclusions

Our observations highlight the need to take into account that any patient may progress to severe disease because of the influence of their genetic background. Therefore, influenza mortality prevention strategies should consider universal vaccination. The *TNF* rs361525 GA genotype is associated with greater disease severity. SNPs in *IL6* are associated with susceptibility to developing influenza A (H1N1) infection. In addition, the *IL6* rs2069869 GG is associated with mortality from influenza A (H1N1) infection in the presence of diabetes. IL-6 levels are increased in both obese pA/H1N1 and ILI patients compared with AHCs, but greater than an obese patient alone. Strikingly, non-obese pA/H1N1 patients presented much higher IL-6 levels than did obese patients. Influenza A (H1N1) virus infection and its severity are determined by the genetic characteristics of the individual, especially among genes that regulate inflammation.

## Supporting Information

S1 Table Genetic information to identify each of the SNPs studied. (PDF)

S2 Table Allele frequencies and associations with susceptibility and risk of severe influenza A (H1N1) infection. (PDF)

S3 Table Association between different polymorphisms and influenza severity. (PDF)

## Abbreviations

AHC: Asymptomatic healthy contacts
AMV: Assisted mechanical ventilation
ARDS: Acute respiratory distress syndrome
BMI: Body mass index
BUN: Blood urea nitrogen
CCL1: Chemokine (C-C motif) ligand 1
CDC: U.S. Centers for Disease Control and Prevention
CI: 95% confidence interval
CPK: Creatine phosphokinase
CRP: C-reactive protein
GLR: Global lethal rate
HLA: Human leukocyte antigen
ICU: Intensive care unit
IFN: Interferon
IL: Interleukin
ILI: Influenza-like illness
LDH: Lactate dehydrogenase
LTA: Lymphotoxin α
OR: Odds ratio
PaO_2_: Partial pressure of oxygen in arterial blood
SNP: Single nucleotide polymorphism
RT-PCR: Real-time PCR
TNF: Tumor necrosis factor
WHO: World Health Organization
