# Supplementary material for: TNF, IL6, and IL1B Polymorphisms Are Associated with Severe Influenza A (H1N1) Virus Infection in the Mexican Population
Source: PLoS One. 2015 Dec 14;10(12):e0144832. doi: 10.1371/journal.pone.0144832 (PMC4682834; doi:10.1371/journal.pone.0144832)
Supplement: S1 Table — (PDF) [file pone.0144832.s001.pdf]

Supplementary table 1. Genetic information to identify each of the SNPs studied.

Gene	SNP	Location			Alleles	
		Chr †	Gene ‡	Region	Change	Ancestral
TNF	rs361525	31651080	4752	(-238) Promoter	G/A	G
	rs1800629	31651010	4682	(-308) Promoter	G/A	G
	rs1800750	31542963	4614	(-376) Promoter	G/A	G
LT	rs909253	31540313	5438	(+252) Intronic	T/C	T
IL1B	rs16944	113594867	4490	(-511) Promoter	G/A	A
	rs3136558	113591275	8082	Intronic	T/C	T
IL6	rs1818879	22772727	10962	3'UTR	G/A	G
	rs2069840	22768572	6807	Intronic	C/G	C
	rs2066992	22768249	6484	Intronic	G/T	G
CCL1	rs2282691	32688309	---	Intronic	T/A	T
IL8	rs2227307	74606669	5447	(+396) Intronic	T/G	T

Fount: The information was obtained from ncbi.nih.gov. Abbreviations:Chr: Chromosome.
