# Supplementary material for: TNF, IL6, and IL1B Polymorphisms Are Associated with Severe Influenza A (H1N1) Virus Infection in the Mexican Population
Source: PLoS One. 2015 Dec 14;10(12):e0144832. doi: 10.1371/journal.pone.0144832 (PMC4682834; doi:10.1371/journal.pone.0144832)
Supplement: S2 Table — (PDF) [file pone.0144832.s002.pdf]

Supplementary table 2. **Allele frequencies and associations with susceptibility and risk of severe influenza A (H1N1) infection.**

Gene/SNP	Allele	pAH1N1 AF (%)	ILI AF (%)	AHC AF (%)	pAH1N1 *vs.* AHC p	pAH1N1 *vs.* AHC OR	pAH1N1 *vs.* AHC 95% CI	pAH1N1 *vs.* ILI p	pAH1N1 *vs.* ILI OR	pAH1N1 *vs.* ILI 95% CI
TNF										
rs361525	G	91.7	93.7	93.5						
	A	8.3	6.1	6.7						
rs1800629	G	96.5	94.6	96.2						
	A	3.4	5.3	3.8						
rs1800750	G	97.9	98.5	98.5						
	A	2.1	1.5	1.5						
IL1B										
rs16944	A	57.3	48.5	53.3				4.80E-02	0.7	0.50-0.98
	G	42.6	51.5	46.6						
rs3136558	T	58.7	53.1	61.8						
	C	41.3	46.9	38.2						
IL6										
rs18181879	G	46.1	53.3	45.9						
	A	53.9	46.7	54.1						
rs2069840	C	70.8	65.1	69.3						
	G	29.2	34.9	30.7						
rs2066992	G	82.7	79.5	74.9	7.00E-03	0.62	0.43-0.88			
	T	17.3	20.5	25.1						
CCL1										

rs2282691	T	58.5	52.4	53.2			
	A	41.5	47.6	46.8			
IL8							
rs2227307	T	63.5	67.4	65.5			
	G	36.5	32.6	34.5			
LTA							
rs909253	G	64.8	65.1	71.3	4.90E-02	1.34	1.01-1.80
	A	35.2	34.8	28.7			

Estimated associations among allele frequencies to determine the risk of severe influenza A(H1N1) infection (p AH1N1 *vs.* ILI) and disease susceptibility (p AH1N1 *vs.* AHC). Only significant data shown .Abbreviations: pAH1N1: patients with influenza A (H1N1); ILI: patients with influenza-like illness; AHC: asymptomatic healthy contacts; GF: genotype frequency; OR: odds ratio; CI: confidence interval.

.
