# Supplementary material for: TNF, IL6, and IL1B Polymorphisms Are Associated with Severe Influenza A (H1N1) Virus Infection in the Mexican Population
Source: PLoS One. 2015 Dec 14;10(12):e0144832. doi: 10.1371/journal.pone.0144832 (PMC4682834; doi:10.1371/journal.pone.0144832)
Supplement: S3 Table — (PDF) [file pone.0144832.s003.pdf]

Supplementary table S3. Association between different polymorphisms and influenza severity.

Gen/SNP	Genotype	UCI GF (%)	NO UCI GF (%)	P	OR	CI 95%
TNF						
rs361525	GG	54 (83.07)	51 (100)			
	GA	8(12.30)	0	0.007	16.06	(1.53-285.45)
	AA	3(4.61)	0	0.36	6.16	(0.59-131.21)
rs1800629	GG	62(95.38)	49(96.07)			
	GA	3(4.61)	2(3.92)	0.76	1.10	(0.23-5.10)
	AA	0	0	---	----	-----
rs1800750	GG	61(93.84)	49(96.07)			
	GA	3(4.61)	1(1.96)	0.79	2.41	(0.24-23.97)
	AA	1(1.53)	1(1.96)	0.57	0.80	(0.049-13.17)
IL1B						
rs16944	AA	25(39.06)	17(33.33)			
	AG	26(40.62)	27(52.94)	0.42	0.65	(0.28-1.48)
	GG	13(20.31)	7 (13.72)	0.89	1.26	(0.41-3.81)
rs3136558	TT	21(31.34)	18(36.73)			
	TC	31(46.26)	22(44.89)	0.81	1.20	(0.52-2.77)
	CC	15 (22.38)	9(18.36)	0.68	1.42	(0.50-4.03)
IL6						
rs18181879	GG	14(21.53)	10(20.83)			
	GA	30(46.15)	24(50)	0.98	0.89	(0.33-2.36)

	AA	21(32.30)	14(29.16)	0.88	1.07	(0.37-3.08)
rs2069840	CC	30(46.87)	26(50.98)			
	CG	28(43.75)	20(39.21)	0.77	1.21	(0.55-2.64)
	GG	6(9.37)	5(9.80)	0.78	1.04	(0.28-3.80)
rs2066992	GG	44(70.96)	31(60.78)			
	GT	18(29.03)	20(39.21)	0.34	0.63	(0.29-1.38)
	TT	0	0	--	------	----
CCL1						
rs2282691	TT	21(38.88)	23(50)			
	TA	28(51.85)	14(30.43)	0.086	2.19	(0.91-5.24)
	AA	15(27.77)	9(19.56)	0.36	1.82	(0.66-5.04)
IL8						
rs2227307	TT	26(40.62)	20(39.21)			
	TG	32(50)	23(45.01)	0.97	1.07	(0.48-2.36)
	GG	6(9.37)	8(15.68)	0.55	0.57	(0.17-1.93))
LTA						
rs909253	GG	29(44.61)	22(43.3)			
	GA	28(43.07)	23(45.09)	1.00	0.92	(0.42-2.01)
	AA	8(12.30)	6(11.76)	0.77	1.01	(0.30-3.34)

Association between different polymorphisms studied and influenza severity, the analyzed genotype and allele frequencies were compared between hospitalized patients who required admission to the ICU and those who did not require admission. Abbreviations: OR: odds ratio; CI: confidence interval
